# The combined effect of gender and age on post traumatic stress disorder: do men and women show differences in the lifespan distribution of the disorder?

**DOI:** 10.1186/1744-859X-9-32

**Published:** 2010-07-21

**Authors:** Daniel N Ditlevsen, Ask Elklit

**Affiliations:** 1 Department of Psychology and Multidisciplinary Chronic Pain Clinic, Odense University Hospital, Odense, Denmark; 2 National Centre for Psychotraumatology, University of Southern Denmark, Odense, Denmark

## Abstract

**Background:**

The aim of the study was to examine the combined effect of gender and age on post traumatic stress disorder (PTSD) in order to describe a possible gender difference in the lifespan distribution of PTSD.

**Methods:**

Data were collected from previous Danish and Nordic studies of PTSD or trauma. The final sample was composed of 6,548 participants, 2,768 (42.3%) men and 3,780 (57.7%) women. PTSD was measured based on the Harvard Trauma Questionnaire, part IV (HTQ-IV).

**Results:**

Men and women differed in lifespan distribution of PTSD. The highest prevalence of PTSD was seen in the early 40s for men and in the early 50s for women, while the lowest prevalence for both genders was in the early 70s. Women had an overall twofold higher PTSD prevalence than men. However, at some ages the female to male ratio was nearly 3:1. The highest female to male ratio was found for the 21 to 25 year-olds.

**Conclusions:**

The lifespan gender differences indicate the importance of including reproductive factors and social responsibilities in the understanding of the development of PTSD.

## Background

Men and women show differences in the age distribution of post traumatic stress disorder (PTSD) prevalence during their lifespan [1]. Although this is supported by a large and thorough epidemiological study, The National Comorbidity Survey (NCS), the finding is limited by the fact that it only involves participants at the age of 15 to 54 years. This must be regarded as a considerable limitation. The average age of living has been increasing in the modern Western world for more than 200 years [2] and passes far beyond the age of 54. It therefore seems reasonable to include a broader range of age when estimating the lifespan distribution of PTSD among men and women. The latest updates show that men living in a country within the European Union (EU) have a life expectancy of approximately 76 years, whereas, women have a life expectancy of nearly 82 years [3]. Therefore, individuals now live for an increased number of years compared to that of previous generations; however, as a result individuals also have more years in which they can experience traumatic events or be affected by the potentially negative consequences that follow traumatic experiences.

It therefore becomes important to pay attention to the risk of PTSD in relation to different stages in the lifespan. This will aid in the progression of age adjusted assessment and treatment methods as well as improving the individual coping strategies of PTSD. Men and women show differences in the biological aspects of brain development, thus differences in behavioural development throughout the lifespan could influence the way risk factors or trauma exposures are met [4]. Additionally, gender has been found to be an important biological determinant of vulnerability to psychosocial stress [5]. Therefore, focusing on the lifespan distribution of PTSD makes sense when it accounts for gender specific developmental details, and when it combines the effect of gender and age on PTSD.

Gender and age often function as demographic variables in PTSD or trauma research and as such they are both very commonly studied. However, for a great deal of studies neither age nor gender differences are the main area of focus. Numerous gender studies have been conducted with regards to PTSD. The main findings regard the fact that women, although less subjected to potentially traumatic events, develop PTSD more often than men [6-8]. Other studies have reported a twofold increase in PTSD prevalence for women compared to men [9]. Speculations have been made that the increased risk of PTSD among females is due to the higher likelihood of females to experience specific trauma types that appear to be particularly traumatic or PTSD inducing [10]. However, it has been reported that the increased prevalence of PTSD in women remains even when trauma type is controlled for [9]. Indications have been made that different trauma types show variations in the extent of gender differences in PTSD prevalence and as such gender shows variation in its effect on PTSD according to trauma type [11].

Fewer age studies than gender studies are represented in the PTSD literature. Thompson, Norris, and Hanacek [12] examined age differences in the psychological consequences of Hurricane Hugo and found that younger people exhibited the most distress in the absence of a disaster, whereas, middle-aged people exhibited most distress in the presence of a disaster. It is obvious to think that increased levels of distress are coherent with an increased risk of PTSD. Norris *et al*. [13] examined the effects of age on PTSD in a cultural context, and compared the effects of age after similar disasters in three different parts of the world. The findings showed no consistent effect of age on PTSD. Therefore, it was concluded that PTSD depended upon the social, economic, cultural, and historical context of the disaster-stricken setting more than it depended on age. They found inconsistent results among respondents from the USA, Mexico, and Poland, where the most distressed were the middle aged, the young, and the old, respectively. Thus, the age differences in PTSD prevalence tend to show some cultural variance.

In one of the most comprehensive epidemiological studies of PTSD conducted to date, the NCS [1], results concluded both gender and age differences in PTSD. The results pertaining to gender differences concluded that women were approximately twice as likely as men to develop PTSD during their lifetime. This finding has since become well established with subsequent studies reaching similar conclusions [6,9]. Interestingly, Kessler and his colleagues concluded no age differences in lifetime rates of PTSD for males across different age groups. However, for women it was suggested that when age increases PTSD rates tend to decrease [1]. The results showed that combining gender and age leads to further information about the prevalence of PTSD. Among the male participants the prevalence of PTSD was highest from their mid 40s to their mid 50s, whereas, the female participants showed the highest prevalence of PTSD from their mid 20s to their mid 30s. The results regarding PTSD prevalence are shown in Table 1.

**Table 1:** PTSD prevalence estimates from nationally representative studies

	**Kessler *et al*. **[1]		**Creamer and Parslow **[14]	
	
	Male	Female	Male	Female
Age, years:				
15 to 24	2.8	10.3	3.8*	5.9*
25 to 34	5.6	11.2	2.5	4.6
35 to 44	5.0	10.6	2.0	3.1
45 to 54	7.6	8.9	2.2	3.7
55 to 64	NA	NA	2.0	1.5
65+	NA	NA	0.4	0.0
Total	5.0	10.4	2.0	3.2

*The result is only based on data from the age of 18 to 24 years.

NA = not available as this age group was not included in the study; PTSD = post traumatic stress disorder.

From the findings of previous research it appears that men and women have different developmental distributions of PTSD during their lifetime. Although the findings by Kessler and colleagues [1] are interesting in this regard, they are still, as mentioned above, limited by the fact that the study only included data on participants between 15 and 54 years of age. Therefore, the study did not include the age extremities of childhood or late life. The relevance of including childhood in the developmental distribution of PTSD during lifetime may seem superfluous or controversial for several reasons. However, the inclusion of childhood would essentially comprise the basis of comparison among the age groups because of obvious biological and psychological differences between children and adults, which may be regarded as important for the perception of the trauma and coping strategies. The inclusion of the age extremities beyond the age of 54 seems otherwise relevant especially with an increasing tendency for the average person to reach old age.

Another comprehensive epidemiological study based on data from the Australian National Survey of Mental Health [14] included participants beyond the age of 54. This study found incoherence for PTSD prevalence rates and exposure to trauma across the lifespan. Results showed that PTSD prevalence reduced with age for both men and women, whereas, a nearly symmetrically inverted U-shaped curvilinear pattern of lifetime exposure to trauma across the lifespan was found for women and a linear increase in lifetime exposure to trauma was found for men. Both male and female participants above the age of 65 reported negligible rates of PTSD. Women showed a higher level of PTSD prevalence in young age and in adulthood compared to men. This effect was seen until the mid 50s where men started to show a higher PTSD prevalence than women. The results of PTSD prevalence among men and women from the study are shown in Table 1. The findings suggested that the highest rates of PTSD prevalence among both men and women are found between the age of 18 and 24 years and the lowest among older people [14]. However, it is important to note that the study only included participants above the age of 18. Some evidence points to the fact that potentially traumatic events as well as the risk of developing PTSD are as much a part of adolescence as it is part of adulthood [15]. Interestingly, the tendency of PTSD prevalence rates declining from young age to old age follows the clinical picture found for PTSD in Danish normative data for the Millon Clinical Multiaxial Inventory III (MCMI-III) [16]. Here, a linear decrease in PTSD prevalence rates according to age was found. This study also concluded a significantly higher score for women compared to men with regards to PTSD.

The finding of low PTSD prevalence in older people is consistent with some studies [17] but inconsistent with others [18,19]. Maercker *et al*. [18] found a substantially higher prevalence of PTSD among participants in the age range of 60 to 93 years compared to the participants below 60 years of age. Thus, the results showed a linear increase in the prevalence of PTSD. However, the increase in prevalence of PTSD among older people could to a large extent be explained by World War II trauma, making the results interesting but also less representative and comparable to populations from non-World War II countries or countries less involved in the war. Elklit and O'Connor [20] examined the occurrence of PTSD in a Danish population sample of older people who had been bereaved. They found that 27% met all four core criteria for PTSD 1 month after losing their spouse; this number was reduced to 17% 6 months after the loss. Findings showed that an additional 16% met a subclinical level of PTSD (missing one criterion) 1 month after the loss. This number had increased to 28% after 6 months. The study did not include gender-related findings. However, elsewhere suggestions have been made that increased age is a bigger risk factor of PTSD for men than it is for women [21], and that younger age significantly predicts PTSD in women but not in men [22]. This is concordant with findings indicating that the mean onset age of PTSD is higher among men than among women [23].

Although the existence of a combined effect of gender and age on PTSD rates is implied by various studies, the results are ambiguous and the differences in lifespan distribution of PTSD among men and women remain unclear. It is the aim of the present study to expand previous research by including a larger number of participants 55 or older and examining the differences in lifespan distribution among men and women, respectively, along with the possible combined effect of gender and age on PTSD prevalence in order to clarify the extent and consequences of such an effect. Knowledge of the lifespan distribution of PTSD could contribute to the aetiology or phenomenology of PTSD. Furthermore, knowledge of such could be beneficial in relation to the assessment or treatment of PTSD. With the previous findings in the PTSD literature in mind, we find it relevant to examine the following hypotheses concerning the lifespan distribution of PTSD prevalence: (a) the lifespan distribution of PTSD will be different for men and women; (b) women will at all ages show a higher prevalence of PTSD than men; (c) men will show their highest level of PTSD prevalence later in life compared to women.

## Methods

### Procedure and participants

The criteria for including studies were: (a) the study included both male and female participants; (b) the Harvard Trauma Questionnaire (HTQ) was used for assessment in the study and thus could be a measure of comparison for the included studies. All studies that did not fulfil the abovementioned criteria were excluded from the study analysis. In addition, the participants (a) should have notified their gender; (b) be between 13 and 80 years of age; and (c) have given full information on the HTQ. Participants who did not fulfil these criteria were excluded.

Two sets of data were made for analysis. A total sample, which included participants from all the studies both epidemiological population samples and convenience samples of different trauma events, and a trauma sample, including only the participants from the convenience samples of different traumatic events within five trauma types; disasters and accidents, loss, malignant disease, non-malignant disease, and violence.

The data for the total sample consequently consisted of data from 25 different studies of trauma and PTSD that were conducted between 1996 and 2008 (Table 2). The final sample was composed of 6,548 participants, 2,768 (42.3%) men and 3,780 (57.7%) women. The age distribution of the participants ranged from 13 to 80 years of age. Of the included studies, 20 were carried out in Denmark, 4 in Iceland, and 1 in the Faroe Islands.

**Table 2:** Convenience and epidemiological samples included in the present study

Category	Male	Female	Total
Disaster and accident:			
Earthquake victims	33	40	73
Explosion affected residents	226	235	461
Rescue personnel dealing with explosion	397	28	425
Whiplash victims	296	1,131	1,427
Violence:			
Assault victims	138	50	188
Knife homicide at a Danish gymnasium	107	172	279
Robbery victims	20	34	54
Malignant or severe disease:			
Families with chronically ill children	32	53	85
Parents of chronically ill children	147	312	459
Non-malignant disease:			
Adolescent and young adults surviving childhood cancer	19	25	44
Cleft surgery patients	18	4	22
Overweight persons	15	141	156
Paraplegics	147	69	216
Parents of prematurely born children	18	40	58
Stutterers	22	6	28
Loss:			
Older people who have been bereaved (pilot study)	20	38	58
Older people who have been bereaved (new study)	248	314	562
Parents who have lost an infant (hospital)	44	55	99
Parents who have lost an infant (parent association)	264	353	617
Youth samples:			
Danish national youth probability sample	145	132	277
Faroese youth population total sample	217	242	459
Icelandic national youth probability sample	73	80	153
Students:			
Social and Health Care College Students	37	83	120
Others:			
Control group from the study of parents who have lost an infant	21	25	46
Trauma clients in treatment	64	118	182
Total	2,768	3,780	6,548

The data for the trauma sample consequently consisted of data from 17 different convenience samples of trauma and PTSD. The final sample consisted of 4,998 participants, 2,039 (40.8%) men, and 2,959 (59.2%) women. The age distribution of the participants ranged from 13 to 80 years of age. The frequency of the 13 to 15 year olds was low in the trauma sample.

For both the total sample and the trauma sample the participants were divided into 14 different age groups with a 5-year span in age for analysis except the age group of 13 to 15 year olds, which only had a 3-year span in age. Three of the included studies were undertaken in Iceland, and the others were completed in Denmark.

All studies included met the ethical guidelines for Nordic psychologists. Studies within the hospital sector were approved by a regional Helsinki committee.

### Measures

The questionnaires used for measurement varied between the individual studies. All questionnaires in the selected studies requested data about gender and age of the participants. The HTQ [24] was used in a Danish, Icelandic or Faroese edition. The HTQ estimates PTSD diagnosis according to the *Diagnostic and Statistical Manual of Mental Disorders, fourth edition *(DSM-IV) [25] and at the same time it measures the severity of PTSD symptoms. The HTQ-IV hereby permits a dichotomous assessment of PTSD. The HTQ originally contained 30 items based on the 3 subscales of PTSD concerning a potentially distressing event. The answers are scored on a four-point Likert scale (1, 'not at all'; 2, 'a little'; 3, 'quite a bit'; 4, 'all the time'). Only scale items above or equal to 3 on the HTQ were considered for a PTSD diagnosis. In all the included studies an item was added or regarding feelings of guilt for something done or omitted. Some studies also divided item 16 (sudden emotional or physical reactions when reminded of the incident) into two questions. However, this additional item was not included in the HTQ total scores used for analysis in the present study, giving a total of 31 items with a possible total HTQ score in the range of 0 to 124. A total of 16 items were related to the 3 subscales of PTSD in DSM-IV: avoidance (7 items), re-experiencing (4 items), and arousal (5 items). Mollica *et al*. [24] have reported good internal consistency, test-retest reliability, and concurrent validity. The HTQ has been used extensively in the Nordic countries [26].

### Statistical analyses

Data were analysed using SPSS V.17.0 (SPSS, Chicago, IL, USA). Statistical tests included descriptive analyses performed on the data using mean scores, standard deviation (SD), and percentages. One-way analyses of variance (ANOVAs) with descriptive statistics were performed to compare the independent variables of gender and age, and the continuous dependent psychometric variable of the HTQ total score. Both the HTQ mean score as well as a categorical PTSD score were ranked by age groups. Both the HTQ mean scores as well as the categorical PTSD scores can be seen as a way to estimate the vulnerability to PTSD. The dimensional and categorical results of PTSD were both ranked by age groups in order to find the estimated distribution of PTSD prevalence according to age.

## Results

### Total sample

**Figure 1:**
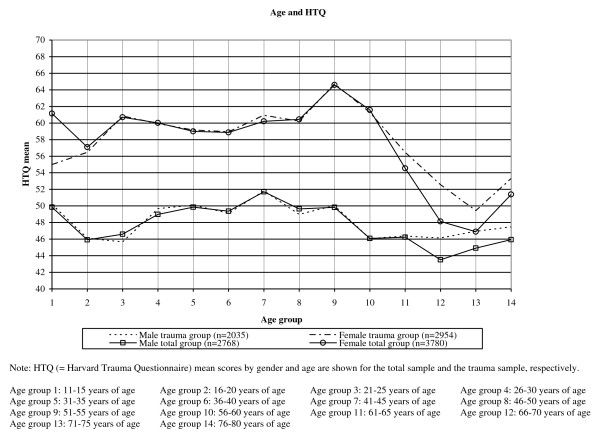
**Lifespan distribution of post traumatic stress disorder (PTSD) based on Harvard Trauma Questionnaire part IV (HTQ) mean scores**.

Ages ranged from 13 to 80 years. For men the mean age was 37.5 years (SD = 17.9) and for women it was 38.1 years (SD = 16.5). Of the participants, 21.3% (n = 1,395; 13% of the men and 27.4% of the women) suffered from PTSD. Women had higher total HTQ scores than men at all ages (*F*_(1, 6,547) _= 532.5; *P *< 0.000). The total HTQ score was 48.57 (SD = 17.26) for men and 59.00 (SD = 18.63) for women. Women showed higher scores for the subscales re-experience, avoidance, and arousal at all ages. Except for the age 71 to 75 years, which showed a higher score for men (mean = 10.86, SD = 3.93) compared to women (mean = 10.63, SD = 3.53) for the subscale avoidance. The mean score of re-experience was 6.89 (SD = 2.66) for men and 8.36 (SD = 2.90) for women. The mean score of avoidance was 10.98 (SD = 4.54) and 12.98 (5.01) for men and women, respectively. For arousal men showed a mean score of 9.16 (SD = 3.92) and women a mean score of 11.91 (SD = 4.20). All subscale gender differences were significant (all F values >275; all *P *values < 0.000). The mean and SD values for the 14 age groups can be seen in Table 3. The HTQ mean scores for these 14 groups are also illustrated in Figure 1 for men and women, respectively.

**Table 3:** Comparison of the different age groups by gender in the total sample

Age, years	Men				Women				Total	
	
	N	PTSD, n	PTSD, %	HTQ total (SD)	N	PTSD, n	PTSD, %	HTQ total (SD)	PTSD, %	HTQ total (SD)
13 to 15	441	50	11.3	49.85 (17.56)	463	144	31.1	61.15 (20.59)	21.5	55.64 (19.98)
16 to 20	217	16	7.4	45.93 (13.56)	237	50	21.1	57.11 (16.47)	14.5	51.76 (16.13)
21 to 25	162	19	11.7	46.60 (17.32)	178	60	33.7	60.69 (20.11)	23.2	53.98 (20.08)
26 to 30	230	34	14.8	48.98 (16.50)	378	99	26.2	60.01 (18.20)	21.9	55.84 (18.24)
31 to 35	305	44	14.4	49.86 (18.31)	500	133	26.6	59.00 (19.98)	22.0	55.53 (19.85)
36 to 40	317	46	14.5	49.38 (19.06)	507	123	24.3	58.87 (18.20)	20.5	55.22 (19.09)
41 to 45	247	45	18.2	51.71 (20.84)	413	119	28.8	60.20 (19.10)	24.8	57.02 (20.18)
46 to 50	214	34	15.9	49.64 (19.63)	289	73	25.3	60.45 (17.70)	21.3	55.85 (19.28)
51 to 55	159	25	15.7	49.84 (16.91)	257	110	42.8	64.60 (17.17)	32.5	58.96 (18.50)
56 to 60	117	16	13.7	46.09 (14.76)	146	57	39.0	61.56 (17.60)	27.8	54.68 (18.09)
61 to 65	72	7	9.7	46.24 (14.27)	80	22	27.5	54.54 (16.95)	19.1	50.61 (16.22)
66 to 70	127	9	7.1	43.51 (10.22)	153	21	13.7	48.12 (13.07)	10.7	46.03 (12.07)
71 to 75	103	7	6.8	44.91 (11.20)	116	12	10.3	46.91 (11.25)	8.7	45.97 (11.25)
76 to 80	57	7	12.8	45.95 (12.59)	63	13	20.6	51.38 (14.37)	16.7	48.80 (13.77)
All ages	2,768	359	13.0	48.57 (17.26)	3,780	1,036	27.4	59.00 (18.63)	21.3	54.59 (18.78)

Mean (SD) values for the 14 age groups are shown for men and women.

HTQ = Harvard Trauma Questionnaire part IV; PTSD = post traumatic stress disorder.

The highest prevalence of PTSD for men (18.2%) was found in the age group of 41 to 45 years while women showed their highest prevalence of PTSD (42.8%) in the age group of 51 to 55 years. The lowest prevalence of PTSD was found at the age of 71 to 75 years for both men (6.8%) and women (10.3%). The total HTQ score was highest at the age of 41 to 45 years for men (mean = 51.71, SD = 20.84) and at the age of 51 to 55 years for women (mean = 64.60, SD = 17.17). The lowest level of total HTQ score was found at the age of 66 to 70 years for men (mean = 43.51, SD = 10.22) and at the age of 71 to 75 years for women (mean = 46.91, SD = 11.25).

A two-way between-groups analysis of variance was conducted to explore the impact of gender and age (based on 5-year spans) on levels of PTSD, as measured by the HTQ. There was a statistically significant main effect for age (*F*_(13, 6,535) _= 12.1; *P *< 0.000); however, the effect size was small (partial eta^2 ^= 0.024). The main effects for gender (*F*_(1, 6,547) _= 333.2; *P *< 0.000) and the interaction effect for gender × age (*F*_(13, 6,535) _= 3.1; *P *< 0.000) were also significant; the effect sizes were, however, also small (partial eta^2 ^= 0.049 and 0.006, respectively). *Post hoc *comparisons using the Tukey B test revealed that the male 41 to 45 years age sample and the female 51 to 55 years age sample were significantly higher in HTQ total scores than the 66 to 70 and the 71 to 75 samples for both genders. The other age groups did not differ significantly from each other.

### Trauma sample

Ages ranged from 13 to 80 years. The mean age for men was 40.7 years (SD = 15.1) and for women it was 40.6 years (SD = 14.1). Of the participants, 21.9% (n = 1,091; 13.8% of the men and 27.5% of the women) suffered from PTSD. At all ages, women showed higher levels of total HTQ score. The total HTQ score for men was 48.71 (SD = 17.57). For women, the total HTQ score was 59.29 (SD = 18.30). The mean and standard deviation values for the trauma sample can be seen in Table 4 and the total HTQ score for the 14 age groups are illustrated in Figure 1 for men and women, respectively.

**Table 4:** Comparison of the different age groups by gender in the trauma sample

Age, years	Men				Women				Total	
	
	N	PTSD, n	PTSD, %	HTQ total (SD)	N	PTSD, n	PTSD, %	HTQ total (SD)	PTSD, %	HTQ total (SD)
13 to 15	5	1	20.0	50.20 (9.26)	9	3	33.3	55.00 (15.35)	28.6	53.29 (13.31)
16 to 20	195	15	7.7	46.11(13.63)	219	43	19.6	56.47 (16.28)	14.0	51.59 (15.94)
21 to 25	137	15	10.9	45.66 (17.46)	136	45	33.1	60.84 (20.53)	22.0	53.22 (24.48)
26 to 30	202	32	15.8	49.67 (17.13)	335	86	25.7	59.91 (17.83)	22.0	56.06 (18.25)
31 to 35	275	40	14.5	50.09 (18.68)	468	123	26.3	59.16 (19.87)	21.9	55.80 (19.92)
36 to 40	291	39	13.4	49.10 (18.94)	472	115	24.4	58.93 (18.36)	20.2	55.18 (19.18)
41 to 45	235	43	18.3	51.81 (21.17)	385	113	29.4	60.39 (19.29)	25.2	57.14 (20.44)
46 to 50	206	30	14.6	48.98 (19.31)	278	65	23.4	60.21 (17.81)	19.6	55.43 (19.26)
51 to 55	153	25	16.3	50.02 (17.17)	242	104	43.0	64.73 (17.32)	32.7	59.03 (18.67)
56 to 60	116	16	13.8	46.08 (14.83)	137	54	39.4	61.28 (17.75)	27.7	54.31 (18.11)
61 to 65	58	6	10.3	46.36 (13.40)	55	18	32.7	56.45 (16.52)	21.2	51.27 (15.77)
66 to 70	58	6	10.3	46.14 (10.52)	93	19	20.4	52.57 (13.92)	16.6	50.10 (13.07)
71 to 75	63	6	9.5	46.94 (11.92)	72	10	13.9	49.43 (11.75)	11.9	48.27 (11.85)
76 to 80	41	6	14.6	47.46 (13.38)	53	13	24.5	53.30 (14.34)	20.2	50.76 (14.16)
All ages	2,035	280	13.8	48.71 (17.57)	2,954	811	27.5	59.29 (18.30)	21.9	54.97 (18.74)

Mean (SD) values for the 14 age groups are shown for men and women.

HTQ = Harvard Trauma Questionnaire part IV; PTSD = post traumatic stress disorder.

The prevalence of PTSD was highest among men (18.3%) at the age of 41 to 45 years. For women the highest prevalence (43.0%) was found for the age 51 to 55 years. The lowest PTSD prevalence for men (7.7%) was seen at the age of 16 to 20 years and for women (13.9%) it was seen at the age of 71 to 75 years. The mean total HTQ score was highest at the age of 41 to 45 years for men (51.81; SD = 21.17) and at the age of 51 to 55 years for women (64.73; SD = 17.32). It was found to be lowest at the age of 21 to 25 and 71 to 75 years for men (45.66; SD = 17.46) and women (49.43; SD = 11.75), respectively. The PTSD prevalence in both the total sample and the trauma sample was twofold higher among women than among men. The highest difference in PTSD prevalence between men and women in the total sample was found for the 21 to 25-year-olds who showed a nearly threefold increase in prevalence among women compared to men. The smallest gender difference in PTSD prevalence was found for the 71 to 75-year-olds who showed a 1.5-fold higher prevalence among women than among men. The trauma sample showed similar results but here the highest difference in PTSD prevalence between men and women was found for the 61 to 65 years sample who showed a more than threefold larger prevalence among women compared to men. For the 21 to 25 years sample a threefold difference was also seen. The smallest difference in PTSD prevalence between men and women was found for the 71 to 75 years sample just like it was seen in the total sample. In the trauma group, the 71 to 75 years sample also showed a ratio of 1 to 1.5 between men and women.

### Ranking of age groups from the total sample

The results showed some differences in the rank of age groups due to a dimensionally (HTQ) or a categorically (PTSD%) estimated PTSD prevalence. The rankings of the age groups from the total sample can be seen in Table 5. The highest rank for women by both PTSD percentages and HTQ scores was found for women at the age of 51 to 55 years. For men the highest rank for both HTQ scores and PTSD percentages were identical as the age group of the 41 to 45-year-olds was found to top both ranking lists.

**Table 5:** Rank of age groups in the total sample by PTSD and HTQ total score.

Rank	Men				Women				Total			
	
	Age, years	PTSD, %	Age, years	HTQ total (SD)	Age, years	PTSD, %	Age, years	HTQ total (SD)	Age, years	PTSD, %	Age, years	HTQ total (SD)
1	41 to 45	18.2	41 to 45	51.71 (20.84)	51 to 55	42.8	51 to 55	64.60 (17.17)	51 to 55	32.5	51 to 55	58.96 (18.50)
2	46 to 50	15.9	31 to 35	49.86 (18.31)	56 to 60	39.0	56 to 60	61.56 (17.60)	56 to 60	27.8	41 to 45	57.02 (20.18)
3	51 to 55	15.7	13 to 15	49.85 (17.56)	21 to 25	33.7	13 to 15	61.15 (20.59)	41 to 45	24.8	46 to 50	55.85 (19.28)
4	26 to 30	14.8	51 to 55	49.84 (16.91)	13 to 15	31.1	21 to 25	60.69 (20.11)	21 to 25	23.2	26 to 30	55.84 (18.24)
5	36 to 40	14.5	46 to 50	49.64 (19.63)	41 to 45	28.8	46 to 50	60.45 (17.70)	31 to 35	22.0	13 to 15	55.64 (19.98)
6	31 to 35	14.4	36 to 40	49.38 (19.06)	61 to 65	27.5	41 to 45	60.20 (19.10)	26 to 30	21.9	31 to 35	55.53 (19.85)
7	56 to 60	13.7	26 to 30	48.98 (16.50)	31 to 35	26.6	26 to 30	60.01 (18.00)	13 to 15	21.5	36 to 40	55.22 (19.09)
8	76 to 80	12.3	21 to 25	46.60 (17.32)	26 to 30	26.2	31 to 35	59.00 (19.98)	46 to 50	21.3	56 to 60	54.68 (18.09)
9	21 to 25	11.7	61 to 65	46.24 (14.27)	46 to 50	25.3	36 to 40	58.87 (18.20)	36 to 40	20.5	21 to 25	53.98 (20.08)
10	13 to 15	11.3	56 to 60	46.09 (14.76)	36 to 40	24.3	16 to 20	57.11 (16.47)	61 to 65	19.1	16 to 20	51.76 (16.13)
11	61 to 65	9.7	76 to 80	45.95 (12.59)	16 to 20	21.1	61 to 65	54.54 (16.95)	76 to 80	16.7	61 to 65	50.61 (16.22)
12	16 to 20	7.4	16 to 20	45.93 (13.56)	76 to 80	20.6	76 to 80	51.38 (14.37)	16 to 20	14.5	76 to 80	48.80 (13.77)
13	66 to 70	7.1	71 to 75	44.91 (11.20)	66 to 70	13.7	66 to 70	48.12 (13.07)	66 to 70	10.7	66 to 70	46.03 (12.07)
14	71 to 75	6.8	66 to 70	43.51 (10.22)	71 to 75	10.3	71 to 75	46.91 (11.25)	71 to 75	8.7	71 to 75	45.97 (11.25)

The ranking of age groups is shown in descending order.

HTQ = Harvard Trauma Questionnaire part IV; PTSD = post traumatic stress disorder.

## Discussion

### PTSD prevalence

The PTSD prevalence in the total sample of 21.3% is quite high compared to findings from previous epidemiological studies [1,6]. However, the result from the present study is based on a large number of convenience samples that have been shown to result in increased prevalence rates for PTSD compared to epidemiological studies [9]. The PTSD prevalence from the trauma sample in the present study does not show a significantly different PTSD prevalence (21.9%) from that found in the total sample. Either the difference in PTSD prevalence between epidemiological and convenience samples is not as distinct as previously assumed or more likely, it is due to the overlap between the total sample and the trauma sample. However, the actual prevalence percentages in the samples are not of key interest as the variation due to gender and age on PTSD prevalence is the objective of the present study. Here, the distribution of epidemiological or convenience samples and the large number of the latter are not likely to affect the results to the same extent. It has been suggested that the gender difference in PTSD prevalence is approximately the same for epidemiological samples and convenience samples [9].

### Gender differences

A 2:1 female/male PTSD ratio was found for both the total sample and the trauma sample, which is consistent with the well established finding of an approximately twofold higher PTSD prevalence among women compared to men [1,6]. In the total sample the overall PTSD prevalence for men is 13% and for women it is 27.4%. However, the female/male PTSD ratio showed some fluctuation between age groups. It also showed variation from the total sample to the trauma sample. The highest female/male PTSD ratio was 3:1 in both samples but the highest ratio was found for a different age group in the total sample (21 to 25 years) than in the trauma sample (61 to 65 years). The age group of 71 to 75 years showed the lowest female/male PTSD ratio (1:1.5) in both samples.

Women were found to score higher on the HTQ in both samples. These findings are consistent with previous findings that also pointed out gender differences for the HTQ [15]. The gender difference in the mean scores of the HTQ is highest for the age groups of 21 to 25 years and 51 to 55 years and smallest for the 71 to 75 years. The results from the present study show that men peak in total HTQ scores a decade sooner than women (41 to 45 years and 51 to 55 years, respectively). Additionally, both men and women seem to be more resistant towards PTSD at old age than earlier in their lives, which is consistent with some previous findings [17] but inconsistent with others [18,19].

Some arguments have been made that the increased PTSD prevalence among women is due to a report bias because men tend to under-report and women tend to over-report symptoms of PTSD [27]. Some of the variance has also been suggested to be due to the social expectancy related to the male and female gender role. Where women are expected to be vulnerable, men are expected to be tough and more resilient to trauma [9]. In relation to the lifespan distribution of PTSD it is possible that some of the noticeable features in the prevalence of PTSD are caused by gender roles, life course expectations, or neurobiological developmental changes as well as by variations in trauma exposure.

### PTSD prevalence and young age

Adolescence has been described as being concerned with identity formation and with the task of developing a sense of self-continuity [28,29], which could contribute to the effects seen in the female and male patterns regarding the age groups of 13 to 15 year-olds, and 16 to 20 year-olds. Both the increased starting point for the early adolescents, as well as the following decrease in PTSD vulnerability for late adolescence, may, to some extent, be caused by identity-related issues.

The early 20s are for women characterised by an increased HTQ score. This is consistent with previous trauma and PTSD-related findings [1,7] that demonstrated an increased risk of PTSD among women in their late teens and early 20s compared to those women at younger age. A long period of adulthood from the 20s to the 40s seems to be characterised by a relatively stable level of HTQ scores which indicates that the vulnerability to PTSD is present and somewhat constant for adult women, despite the fact that this period in life is known to hold many life changing moments such as, getting married, starting a family, choosing a career, and so on. Perhaps herein is a great part of the explanation. Frequent changes and individual development happening in tune with the modern female gender role throughout most of the period brings meaning and life satisfaction to each individual woman. However, the vulnerability rises to its peak around the early 50s where the risk of PTSD is significantly high. This deviates from the previous level of HTQ scores and hereby indicates a significant change in the life course caused by neurobiological or other factors.

### Midlife crisis

Fluctuations in the reproductive hormones across menstrual phase and reproductive state in women have been found to influence the sympathetic system reactivity [30]. An increased level of activity in the sympathetic or noradrenergic systems has additionally been found to be present in men and women with PTSD. It is, therefore, plausible that exposure to traumatic stress during different phases of the menstrual or reproductive cycles could influence the vulnerability to PTSD due to different effects at a neurobiological level. Menopausal women have shown increased cardiovascular and epinephrine responses to mental stress compared to premenopausal women [31], and PTSD symptoms have been associated with ambulatory cardiovascular functioning in postmenopausal women [32]. This might provide a neurobiological explanation for the increased HTQ scores found for women at the age of 51 to 55 years in the present study. The age of 51 to 55 years is equivalent to the age of menopause. Changes in reproductive ability, hormonal levels, and sympathetic responses are some of the likely changes that happen along the transition from a premenopausal to a postmenopausal woman. The changes are, therefore, not merely neurobiological but also involve potential changes in self-perception, social participation, world beliefs, and adaptation to social gender roles. These changes might also add to an increased stress level or a greater vulnerability to PTSD.

For men a different pattern is seen in adulthood. The male pattern is characterised by a steady almost linear increase in HTQ scores, which begins in the late teens or early 20s and lasts to the early 40s, where the HTQ scores for men peaks. It is conceivable that the gradual rise in the risk of PTSD happens concordantly with a gradual change in the male gender role from being free and able to do as they please to being tied up with work and family responsibilities, resulting in a life with less autonomy and potentially more stress. The phenomena of male midlife crisis might, to some extent, influence the results in HTQ scores found in men.

### PTSD prevalence in old age

A distinct decrease in HTQ scores is seen for women after the 50s and the lowest level is found for women in their late 60s or early 70s. For men a decrease in HTQ scores is also seen towards old age. After the 40s men show a gradual fall in the risk of PTSD. The lowest potential risk of PTSD is thus found for men in their late 60s. Old age has been considered to deal with the acceptance of earlier experiences in life and the fact that death is more imminent than earlier [33]. According to Erikson [34] old age is concerned with the psychosocial crisis of ego integrity versus despair. If the crisis is resolved favourably, ego integrity, wisdom, and life satisfaction is reached [33,34]. This could in fact be part of the reason for the decreased risk of PTSD seen in the 50s and 60s for men and in the late 50s, 60s, and early 70s for women. Some suggestions have additionally been made that a decline in self-occupation, an increase in time spent in quiet reflection, and a decreased interest in superfluous social interactions also are characteristic of old age [35]. Satisfaction with the life led, wisdom in retrospect, and the acceptance of a forthcoming death without fear may very likely affect coping strategies and resilience to PTSD in a positive way. However, this is challenged by the results for both men and women in the present study. Thus, the risk of PTSD shows a small linear increase from the late 60s to the late 70s for men and from the mid to late 70s for women. It has been suggested that reaching the age of 80 or more involves special challenges and perhaps a new stage in psychosocial development [33,34]. If this is the case the vulnerability to PTSD might also be different and involve special issues at such an old age. The effect might, to a limited degree, be detectable in the results for the 70 or 80-year-olds in the present study and thus explain the final rise seen in the HTQ scores.

### Comparison with previous studies

Kessler *et al*. [1] found that the age group of 45 to 54-year-olds showed the highest risk of PTSD among men. Among women they found it to be between the age of 25 and 34 years. Creamer and Parslow [14] found the highest risk of PTSD to be present between the age of 18 and 24 years for both men and women. When converting our results into comparable 10-year-span age groups the highest risk of PTSD was found between the age of 45 and 54 years among men, and 55 and 64 years among women. The results for men are congruent with the findings by Kessler *et al*. The results for women, however, show a 30-year difference in PTSD peaks between the two studies. However, the age group of 55 to 64-year-olds was not included in the Kessler *et al*. study. The inconsistency with the findings of Creamer and Parslow could be due to methodological or cultural differences.

The findings from the present study describe the effect of gender and age on PTSD prevalence in a Nordic country culture context. The results for effect of age on PTSD prevalence resemble the results found by Norris *et al*. [13] in relation to an American culture context. The total picture of PTSD prevalence (based on HTQ mean score) associated with age shows that the prevalence of PTSD follows a curvilinear picture where middle-aged participants show a higher degree of PTSD than young participants, who again show a higher level of PTSD than older people. The results, thereby, are somewhat similar to the ones found in the US by Norris *et al*. and might reflect how PTSD appears in Western cultures. In contrast, Maercker *et al*. [18] found a higher prevalence of PTSD among participants above the age of 60 years in Germany. This might show that cultural comparison of PTSD prevalence or other psychological measures can be delicate due to, for example, historical, economical, or political reasons.

### Age group ranking

Both the HTQ mean scores as well as the categorical PTSD scores can be seen as a way to estimate the potential risk of PTSD or the vulnerability to PTSD. The dimensional and categorical results of PTSD were both ranked by age groups in order to find the estimated distribution of PTSD prevalence according to age. The results show differences in the rank of age groups due to a dimensionally (HTQ) or a categorically (PTSD%) estimated PTSD prevalence. The rankings of age groups can be seen in Table 5. The highest rank for women by both the PTSD percentages and the HTQ scores is found for women at the age of 51 to 55 years. For men the highest rank for both HTQ scores and PTSD percentages are identical as the age group of the 41 to 45-year-olds is found to top both ranking lists for men. The second and third most PTSD vulnerable age groups according to the HTQ are the 31 to 35 years sample and the 13 to 15 years sample among men and the 56 to 60 years sample and the 13 to 15 years sample among women. For the categorical PTSD prevalence the second and third rank are found for the 46 to 50 years sample and the 51 to 55 years sample among men and the 56 to 60 years and the 21 to 25 years samples among women. Thus, the two types of measurement primarily show differences in the ranking of age groups but some congruent results are found. Recent articles indicate that the future choice of measurement in the DSM will favour the dimensional proportions [36,37]. As indicated by the results from the present study differences are seen between the two types of measurement. Which is preferable to the other is not settled by the present study, but the HTQ score does withhold more information. Thus, the results based on the dimensional approach might be more differentiated. This might add some consideration to the ongoing discussion of the preference of dimensional models rather than categorical measures in the research agenda for the DSM-V [38].

### Limitations of the study

In this study, 25 different studies were included to test the hypothesis that men and women show a difference in age distribution of PTSD prevalence. Possible limitations due to a lack of representativeness in the samples, undetected cohort effects, and biases due to method failure are likely to have influenced the results. However, a great strength of the study is the size of the population by which each age group has reached a certain representative size. All the data has been analysed by retrospective analysis and no contact has been made with any participants in the process of the present study. Possible reporting biases could, therefore, have gone undetected or have been deleted due to ambiguity. A large part (76%) of the total sample consisted of convenience samples. This of course must be viewed as a potential limitation of the study and must be taken into account when interpreting the results of PTSD prevalence.

Another limitation of the study concerns cultural considerations. The present study is based on data gathered in the Nordic countries of Denmark, Iceland, and The Faroe Islands. The results, therefore, must be taken with some consideration when comparing to other countries or areas outside the Nordic region. Norris *et al*. [13] clearly showed that cultural differences are found in the PTSD prevalence rates. Therefore, it is likely that cultural considerations account for some of the variance seen in the present study. It is desirable that the combined effect of gender and age on PTSD is studied in other parts of the world in order to see if the present study has created a precedent for the combined effect of gender and age on PTSD or for the lifespan distribution of PTSD.

### Future research

To conclude on the matter of gender differences in the lifespan distribution of PTSD it would be beneficial to compare the age distribution of PTSD prevalence with the age distribution of trauma exposure in order to find potential discrepancies and in order to clarify the true extent of the vulnerability or risk of PTSD. If controlling for trauma exposure does not indicate that the combined gender and age effect on PTSD is due to increased trauma exposure at certain periods in the male or female life course then the results from the present study demand further research. Thus, the goal of future research would be to verify the presented findings as well as to find possible explanations for these findings. Future research should focus on the construction of usable and representative age groups with inclusion of not only young and adults but also older people in order to describe the entire lifespan of PTSD distribution. The inclusion of participants beyond the age of 80 would touch on something new and concurrently bring diversity into the range of the population examined. Future research should also include an examination of the association of different trauma types in order to find possible exposure biases or other possible effects seen from specific trauma types.

The combined effect of gender and age on PTSD has previously been given little attention in the PTSD literature. However, the results from the present study indicate that it makes sense to consider the combined effect of gender and age on PTSD as it outlines how risk of PTSD or PTSD vulnerability can be seen in a lifetime perspective. The lifespan distribution of PTSD shows that men are most vulnerable to PTSD a decade sooner than women. This difference is of particular interest and needs to be investigated further in future research in order to develop more thorough explanations for the effect.

## Summary

The hypotheses made for the present study are verified by the results found. The first hypothesis, that the lifespan distribution of PTSD will be different for men and women, is supported by the results. Women and men are found to show different lifespan distributions of PTSD. However, some similarities are seen in the fluctuations of HTQ score during the lifespan. Thus, some of the rises and falls in HTQ scores follow the same age pattern for men and women and are only different in terms of an elevated level of scores among women and in the gender-related ranking of age groups. In contrast, the most evident difference consists of the rise in HTQ scores around the early 50s for women, which is simultaneous with a fall in HTQ scores for men. The main differences thus consist of an elevated level of HTQ scores or PTSD prevalence for women compared to men, and a peak in HTQ scores or PTSD prevalence found at different points during the lifespan for men and women, respectively. This hints that verification is also found for the second hypothesis, that women at all ages will show a higher prevalence of PTSD than men. Support for this second hypothesis is found both in terms of dimensionally (HTQ score) and categorically (qualification for PTSD criteria) measured PTSD. Confirmation is not found in the results for the last hypothesis, that men will show their highest level of PTSD prevalence later in life compared to women. The male participants from the present study showed their highest level of PTSD prevalence about a decade sooner in their lifespan compared to the female participants.

## Conclusions

The findings from the present study differ from previous findings regarding the combined effect of gender and age on PTSD prevalence. The results for men show some consistency with previous findings, whereas, the results for women do not. The results show that men are most vulnerable to PTSD at the age of 41 to 45 years, whereas, women are most vulnerable to PTSD at the age of 51 to 55 years. Thus, men and women peak in the risk of PTSD a decade apart from each other during their respective lifespan. The female/male ratio of PTSD prevalence is found to vary between the different age groups. However, at all ages from 13 to 80 years women show a higher level of PTSD prevalence than men.

## Competing interests

The authors declare that they have no competing interests.

## Authors' contributions

AE conceived the study and participated in the design of the study. DND prepared the data file, performed the statistical analysis, and drafted the manuscript. Both authors read and approved the final manuscript.
